# Astrocytes & Astrocyte derived Extracellular Vesicles in Morphine Induced Amyloidopathy: Implications for Cognitive Deficits in Opiate Abusers

**DOI:** 10.14336/AD.2021.0406

**Published:** 2021-09-01

**Authors:** Susmita Sil, Seema Singh, Divya T Chemparathy, Ernest T Chivero, Lila Gordon, Shilpa Buch

**Affiliations:** Department of Pharmacology and Experimental Neuroscience, University of Nebraska Medical Center, Omaha, NE 68198-5880, USA.

**Keywords:** Morphine, astrocytes, amyloids, EVs, HIF-1α

## Abstract

While opiates like morphine play a major role in the pharmacotherapy for the control of pain associated with various diseases, paradoxically, their long-term use is associated with cognitive impairments. Furthermore, morphine administration has been shown to result in neuronal synaptodendritic injury in rodent brains, leading to neurodegeneration. We recently reported the role of astrocytes as contributors of amyloidosis associated with HIV-associated neurological disorders. Herein we hypothesize that morphine could induce astrocytic amyloidosis, which, in turn, could be disseminated to various regions in the brain by astrocyte-derived EVs (ADEVs). In this study we demonstrate brain region-specific up-regulation of astrocytic amyloids in morphine dependendent rhesus macaques. In addition, herein we also demonstrate increased expression of β-site cleaving enzyme (BACE1), APP, and Aβ in human primary astrocytes (HPAs) exposed to morphine. Mechanisms involved in this process included the up-regulation of hypoxia-inducible factor (HIF-1α), its translocation and binding to the promoter of BACE1, leading to increased BACE1 activity and, generation of Aβ 1-42. Gene silencing approaches confirmed the regulatory role of HIF-1α in BACE1 mediated amyloidosis leading to astrocyte activation and neuroinflammation. We next sought to assess whether morphine-stimulated ADEVs could carry amyloid cargoes. Results showed that morphine exposure induced the release of morphine-ADEVs, carrying amyloids. Interestingly, silencing HIF-1α in astrocytes not only reduced the numbers of ADEV released, but also the packaging of amyloid cargos in the ADEVs. These findings were further validated in brain derived EVs (BEVs) isolated from macaques, wherein it was shown that BEVs from morphine-dependent macaques, carried varieties of amyloid cargoes including the cytokine IL-1β. This is the first report implicating the role of HIF-1α-BACE1 axis in morphine-mediated induction of astrocytic amyloidosis, leading, in turn, to neuroinflammation and release of the amyloid cargoes via ADEVs. These findings set the groundwork for the future development of therapeutic strategies for targeting cognitive deficits in chronic opiate users.

Morphine is one of the most potent prescription analgesics among all the opiates that is used extensively in the clinical setting [1, 2]. On, an average 17% or more of Americans have at least one opioid prescription filled, with a mean of 3.4 opioid prescriptions dispensed per patient (www.cdc.gov/drugoverdose/data/prescribing/prescribing-practices.html). As per the CDC, in 2017, there were 58 opioid prescriptions written for every 100 Americans. Infact, from 2007 to 2017, there has been ~2.6-fold increase in opioid overdose deaths (NIH, 2019). Several reports have shown that extensive opioid use leads to complications including addiction, tolerance, cognitive impairment, withdrawal symptoms, memory impairment, depression and severely compromised immune system as well as increased risk of opportunistic infections [3-12]. Furthermore, it has also been reported that patients undergoing short-term hospital stay for surgery are also at an increased risk of long-term dependence on prescription opioids [13]. With the increased morbidity associated with pain-related conditions such as cancer and neuropathies, morphine abuse has escalated globally and has become a serious global problem, leading, in turn, to comorbidity of opioid induced cognitive dysfunction [14]. Approximately, 20-44% of the cancer patients taking morphine develop cognitive decline [14]. In keeping with this, other reports have also shown impairment of prospective memory [12], as well as attention, complex working memory, and episodic memory [15, 16] in long-term opiate users. Emerging evidence suggests that neural circuits underlying opiate use disdorders share some common pathways as those underlying learning and memory.

Alzheimer’s Disease (AD) is a dreaded neurodegenerative disease characterized by the presence of extracellular amyloid plaques and intraneuronal tangles [17, 18], which play a key role in neurodegeneration & cognitive impairment. Amyloid β (Aβ) is a key molecule implicated in the development of AD pathogenesis [19], which is generated from sequential cleavage of the amyloid precursor protein (APP) by two proteolytic enzymes- β- secretase (beta-site APP cleavage enzyme, BACE) and γ-secretase [20, 21]. Dysregulation of the BACE1 enzyme and/ or γ-secretase leads to the production and accumulation of Aβ, thereby contributing to the pathogenesis of AD [22, 23]. In addition to amyloids [24], there are also other reports demonstrating increased deposition of hyperphosphorylated tau in the brain regions (frontal, temporal cortex and locus coeruleus) of opiate users compared to age matched controls [24, 25]. It is likely that use of opiates such as morphine, that has detrimental effects on cognitive functioning, could likely attribute to AD-like brain pathology. There are no reports on this phenomenon and warrant further investigation.

In our recent report we showed that HIV-1 protein Tat induced astrocytic amyloidosis involving the HIF-1α-BACE1-AS axis, leading to the development of AD- like pathology in both HIV-1+ patients and in SIV-infected macaques, that was linked with HIV-associated neurological disorders (HAND) [26]. Based on the premise that HIV-infected patients are also afflicted with substance abuse disorders (SUDs), specifically opiates, we wanted to test whether opiates on their own could also mediate cognitive deficits and if so, what were the mechanisms involved. Furthermore, studies from our group have demonstrated earlier that morphine exposure mediates neuronal synaptodendritic injury in neuronal cultures as well as rodent brains involving the endoplasmic reticulum (ER) stress-autophagy axis, and leading to neurodegeneration [27]. Additionally, several reports have also shown morphine mediated cognitive dysfunctioning both in rodents as well as in patients on opiate therapy [11, 12]. In AD, it is suggested that one of the key causes of cognitive impairment is dysregulation and accumulation of Aβ, happening early in the disease and often preceeding the onset of dementia. More recently, in addition to neurons as the source of Aβ, other cells such as the astrocytes that comprise ~50-70% of the total cell population in the central nervous system (CNS), have also garnered interest as potential contributors of amyloidosis in AD [28, 29]. If indeed these cells also play a role in inducing amyloidosis, this process could translate into a significant added burden to the process of brain amyloidosis. Till date, however, there are no reports on the role of morphine in astrocytic amyloidosis, leading to neuroinflammation which, in turn, could manifest as cognitive impairments.

Recent studies have also shown that endosome-derived multivesicular bodies can function as conduits for transferring intercellular cargoes to extracellular space (ECS) and to neighboring or distant recipient cells [30]. Extracellular Vesicles (EVs) play important roles as cargo-carrying vesicles mediating communication among diverse cell types and tissues [31-33]. Furthermore, the release kinetics of EVs have also shown to be altered in various pathologies [34-38]. EVs have been implicated to play key roles in various neurodegenerative diseases, such as Alzheimer’s Disease [39], Parkinson’s disease [40], Amyotrophic Lateral Sclerosis [41] as well as HIV-associated neurocognitive disorders (HAND) [42]. Additionally, in both homeostatis as well as disease state astrocyte derived EVs (ADEVs) have been shown to play critical roles in modulating cellular functions in the CNS [43, 44]. In AD, EVs carrying amyloid cargoes have been postulated to serve as biomarkers of disease pathogenesis [45] while also playing a role in seeding of amyloids, thus disseminating the disease [46], leading to disease progression and severity [47-49].

Taken together we thus hypothesized that morphine could induce the process of astrocytic amyloidosis leading to neuroinflammation, and these amyloids could be packaged in the EVs, leading to their dissemination in brain areas, thereby correlating with cognitive deficits. In the current study, we report astrocytic amyloidosis in the archival brain tissue of rhesus macaques chronically administered morphine. We also report that the neurotoxic amyloid cargoes were packaged in the brain-derived EVs from macaque brains. Furthermore, dissection of the molecular pathway underlying morphine-mediated induction of amyloids in human primary astrocytes (HPAs) involved up-regulated expression of HIF-1α and its regulation of the BACE1 promoter, leading to increased expression of the cleaved toxic Aβ 1-42 form, ultimately resulting in astrogliosis and neuro-inflammation. Furthermore, we found that these amyloids and neuroinflammatory cargoes were packaged in the ADEVs and released to the ECS, which could contribute to neurodegeneration and neuroinflammation involving the HIF-1α-BACE1 axis. This is the first report describing morphine-mediated induction of astrocytic amyloidosis & its release in EVs involving the HIF-1α-BACE1 axis. HIF-1α can thus be considered as a potential therapeutic target for ameliorating morphine-induced cognitive impairment in patients on chronic opiate therapy.

## MATERIALS AND METHOD

### Reagents

Following antibodies were used for this study. AβmOC64 (ab201060, Abcam, MA, USA), Aβ 1-42 (ab10148,), BACE1 (ab63954, Abcam, MA, USA), APP (ab15272, Abcam, MA, USA) CD63 (ab216130, Abcam, MA, USA), CD9 (ab92726, Abcam, MA, USA), Flotillin (ab133497, Abcam, MA, USA), TSG101 (ab125011, Abcam, MA, USA), Alix (ab275377, Abcam, MA, USA), Calnexin (ab133615, Abcam, MA, USA), Argonuate-2 (ab186733, Abcam, MA, USA), GFAP (G3893, Sigma- Aldrich, MO, USA), β-actin (A5316, Sigma- Aldrich, MO, USA), HIF-1α (NB100-449, Novus Biological Company, CO, USA), goat anti-rabbit (sc-2004, Santa Cruz Biotechnology, TX, USA) and goat anti-mouse (sc-2005, Santa Cruz Biotechnology, TX, USA), Alexa Fluor 488 conjugated goat anti-mouse (A11001, ThermoFisher Scientific, MA, USA), Alexa Fluor 647 conjugated goat anti-mouse (A32728, ThermoFisher Scientific, MA, USA), Alexa Fluor 594 conjugated goat anti-rabbit (A11012, ThermoFisher Scientific, MA, USA).

### Macaques

Archival brain tissues from Indian rhesus macaques (*Macaca mulatta*) were used for this study. All the macaque experiments were conducted according to the protocols approved by the Institutional Animal Care and Use Committee. Macaques were exposed to morphine as described previously [50]. Briefly, 12 weeks of morphine injections (i.m., three times daily) were administered in monkeys (n=4) with an initial dose of 6 mg/kg body weight (b.w.) in the first week, 9 mg/ kg b.w. in the second week followed by 12 mg/ kg b.w. for the remaining 10 weeks. For the control group, monkeys (n=4) received saline three times daily for 12 weeks. At the end of the treatment period of 12 weeks, the animals were sacrificed, and different brain regions were dissected and processed for the further experiments. Brain homogenates were used for assessing the expression levels of various proteins and mRNAs of interest as well as EV isolation. Paraffin fixed sections were used to determine the expression levels of Aβ 1-42, Aβ m0C64 and GFAP immunostaining.

### Cell culture

Human primary astrocytes (HPA) were procured from ScienCell Research Laboratories (1800, Carlsbad, USA) and were grown in astrocyte culture media (1801, ScienCell Research Laboratories, Carlsbad, CA) supplemented with 2% fetal bovine serum (0010, ScienCell Research Laboratories, Carlsbad, CA), astrocyte growth supplement (1852, ScienCell Research Laboratories, Carlsbad, CA) and penicillin-streptomycin solution (0503, ScienCell Research Laboratories, Carlsbad, CA) until 70% confluency at 37°C in a humidified incubator with 5% CO_2_. HPAs were cultured at a density of 0.3 × 10^6^/well (6 well plate) and 0.05 × 10^6^/well (24 well plate) for the experiments with a maximum of 5 passages. Cells were serum-starved prior to exposure to morphine.

### Quantitative real-time PCR (qPCR)

Total RNA was isolated from HPAs using Quick-RNA MicroPrep kit (Zymo Research Corporation, USA, R1055) according to the manufacturer’s instruction. qPCR was performed as described previously [51]. Briefly, 1µg of total RNA was reversely transcribed into complementary DNA using Verso cDNA synthesis kit (Thermo Fisher Scientific, USA, AB1453/B) as per the manufacturer's protocol. qPCR reactions were performed using Taqman probes. The commercial primers for BACE1 (Hs_01121195), APP (Hs_00169098), HIF-1α (Hs_00153153), GAPDH (Hs_002786624), TNF-α (Hs_00174128), IL-1β (Hs_01555410), IL-6 (Hs_00 174131) were purchased from Thermo Fisher Scientific, USA. Relative values were obtained by normalizing averaged CT values to housekeeping control GAPDH and the fold change in the gene expression was calculated by 2^-ΔΔCT^ method.

### Western blotting

Standard western blotting procedure was followed to analyze the protein expressions in samples as described previously [26]. Briefly, proteins were extracted from HPAs as well as brain tissues of experimental macaques using the Mammalian Cell Lysis kit (MCL1-1KT, Sigma- Aldrich, St. Louis, MO, USA) and were quantified by BCA assay using Pierce BCA Protein Assay Kit (23227, Thermo Fisher Scientific, USA) according to the manufacturer’s instruction. The proteins were separated by SDS PAGE and were transferred to PVDF membrane (IPVH00010, Millipore Sigma, MO, USA). Immuno-blotting was performed using specific primary antibodies and horseradish peroxidase linked anti-mouse and anti-rabbit IgGs. The immunocomplex was detected by SuperSignal chemiluminescent substrate (VJ311133, Thermo Fisher Scientific, USA) according to the manufacturer’s instruction. β-actin antibody was used to normalize the protein expression. Images of protein bands were acquired using a digital photo scanner GT-X750 (Seiko Epson Corp) and were quantified using Image J (v1.4.3.67; NIH, Bethesda, MD) software.

### siRNA Transfection

siRNA transfection has been carried out as described previously [52]. In brief, HPAs were seeded in 6-well plates (0.3 × 10^6^ cells per well) and thereafter, incubated overnight at 37°C in a humidified, 5% CO_2_ incubator. Human *HIF-1α* siRNA (sc-35561, Santa Cruz Biotechnology, TX, USA) and human *BACE1* siRNA (sc-37224, Santa Cruz Biotechnology, TX, USA) were used to silence the expressions of HIF-1α and BACE1 expressions respectively in HPAs (0.3 × 10^6^ cells per well). Gene silencing was confirmed by qPCR and Western blotting.

### β secretase activity

HPAs (0.3x10^6^ cells) at 70% confluency were exposed with morphine (0.5 µM) for 24h. After the experimental period, the cells were washed in ice-cold PBS and BACE1 activity was assessed using fluorometric based Beta-Secretase Activity Assay Kit (ab65357, Abcam, MA, USA) according to the manufacturer’s protocol.

### Immunohistochemistry

Formaldehyde fixed paraffin-embedded tissue sections (5 µm) of frontal cortex and basal ganglia of saline and morphine treated macaques were deparaffinized in xylene and re-hydrated in descending grades of ethanol and deionized water. Following antigen retrieval in Tris EDTA buffer (pH, 9.0) and blocking, the tissue sections were probed with primary antibodies Aβ 1-42, Aβ m0C64 and GFAP. Immunostained tissues were detected with respective fluorescent-conjugated secondary antibodies and the images were acquired using Z1 inverted microscope (Carl Zeiss, Thornwood, NY). All the images were analyzed using the AxioVs 40 Version 4.8.0.0 software (Carl Zeiss MicroImaging GmbH) and quantified by Image J (v1.4.3.67; NIH, Bethesda, MD) software.

### Isolation of EVs from astrocytes

Extracellular vesicles (EVs) were isolated from the cell culture supernatant of HPAs by differential centrifugations as described previously [43]. Briefly, conditioned media were harvested from different treatment groups of HPAs and, centrifuged at 300 × g for 10 min and 2000 × g for 10 min to remove cell debris. The supernatants were centrifuged again at 10,000 × g for 30 min and filtered through 0.22-μm filter. Exosomes were concentrated by ultracentrifugation at 100,000 × g for 70 min (Beckman Ti32 rotor; Beckman Coulter, Brea, CA, USA) and exomeres were concentrated by ultracentrifugation at 167,000 × g for 16h [53]. EVs were quantified by NTA analysis using Nanosight NS300 instrument as described previously [43, 54]. EVs were further characterized for the expression of fraction specific markers by Western blotting, atomic force microscopy and transmission electron microscopy.

### Isolation of EVs from macaque brain

For the isolation of EVs from macaque brain, saline and morphine administered macaque brains tissues (1000 mg) were homogenized in Hibernate A medium (10-15ml) containing proteolytic enzyme papain (20 units/mL) for 30 min on gentleMACS Octo Dissociator with heaters (Miltenyi Biotec, USA). Tissue homogenates were centrifuged at 300 x g for 10 min, 2000 x g for 10 min at 4°C to eliminate cell debris. Supernatants were collected and centrifuged at 10,000 x g for 30 min to separate large vesicles. Supernatants were filtered through 0.2 μm filters and the small particle sized EVs were collected through ultracentrifugation at 100,000 x g for 70 min at 4°C. EV pellets were further processed for gradient purification using 5, 10, 20 and 40% iodixanol (Optiprep; Cosmo Bio USA) gradient as described previously [55]. Briefly, gradient was prepared by adding 40% iodixanol (3 ml) at the bottom of the ultracentrifugation tube followed by layers of 20, 10 and 5% of iodixanol (3 ml each) to the top. EV pellets were suspended in 500 µl of 0.25 M sucrose buffer [10 mM Tris, pH 7.4] and were added on the top of the layered iodixanol followed by ultracentrifugation at 100,000 x g in a SW40 Ti rotor for 18 h at 4°C. After centrifugation, 500µl was discarded from the top and 1 ml fractions (F1-F12) were collected containing EVs into different tubes. The EVs were further washed with filtered PBS to remove iodixanol from the samples by ultracentrifugation at 200,000 x g in a SW40 Ti rotor for 2h at 4°C. EVs were quantified by NTA analysis using Nanosight NS300 instrument as described previously [43, 54]. EVs fractions were further characterized for the expression of fraction specific markers by western blotting, atomic force microscopy and transmission electron microscopy.

### Zeta view tracking analysis

Isolated EVs from the brain and HPAs were analyzed by nanoparticle tracking analysis (NTA) using ZetaView Nanoparticle Tracking Analyser (Particle Metrix, Germany) along with the software ZetaView 8.04.02 SP1. Prior to the analysis, the instrument was caliberated using 100 nm polystyrene nanostandard particles and cell quality checking was performed before sample reading. The video was captured at a shutter speed of 100, sensitivity to 85 and frame rate of 30. Size (diameter in nm) and concentration (particles/ml) for each sample was determined by injecting the diluted sample in filterd PBS, with 2 cycles reading at each position. The values obtained from the analyzer were finally calculated by multiplying with the dilution factor.

**Figure 1.**
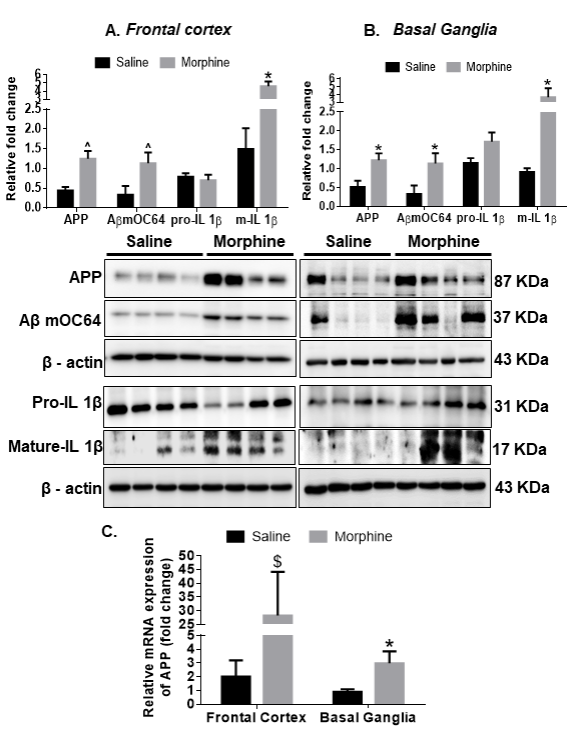
**Amyloidopathy in the brain regions of morphine-dependent macaques**. Representative western blots showing the expression of APP, Aβ mOC64, pro-IL1-β and mature- IL1-β in (A) frontal cortex (FC) and (B) basal ganglia (BG) of saline and morphine dependent macaques. (**C**) qPCR analysis showing expression of APP mRNA in the FC and BG of saline and morphine dependent macaques. *N* = 4 macaques per group. Data are presented as mean±SEM. Student *t* test was used to determine the statistical significance between the groups: $P <0.01, *P< 0.05 versus saline. Abbreviations: APP, Amyloid Precursor Protein, Aβ, amyloid β, IL1-β, interleukin, qPCR, quantitative polymerase chain reaction.

### Electron Microscopy

Negative staining was performed with EV pellets with slight modifications. Briefly, EV pellets (3 ml) were deposited on 200-mesh Formvar-coated copper grids and the membranes were covered for 4-5 minutes for the absorption. The grids were further transferred to uranyl acetate solution for contrast staining. Hereafter, the grids were washed with PBS and excess fluid was blotted with filter paper and allowed to air-dry at room temperature. Imaging was performed using Hitachi H7500 electron microscope (Hitachi, Tokyo, Japan) at 200kV.

### Atomic Force Microscopy

The 1-(3-aminopropyl) silatrane (APS) was functionalized on freshly cleaved mica surface by depositing 500µM APS solution onto the mica and incubating for 30 min with a wet cap on it as described previously [56]. EV samples (20 µl, 8.3 x 10^9^ EVs/ml) were deposited on APS functionalized mica and incubated for 20 min at normal room temperature (RT). The sample was then rinsed with PBS and was subjected to imaging using Asylum Research MFP3D instrument equipped with MSNL ‘E’ cantilevers with a spring constant of 0.1 N/m (Santa Barbara, CA, USA) without allowing the mica surface to get dried.

### Statistical analysis

The grouped data are represented as mean ± SEM. The statistical significance among the multiple experimental groups was determined by one-way ANOVA followed by Bonferroni post hoc test and between two groups was analyzed by student t- test using the GraphPad Prism Sofware (Version 5). Statistical analysis where p value was less than 0.05, 0.01, 0.001 were considered as statistically significant.

**Figure 2.**
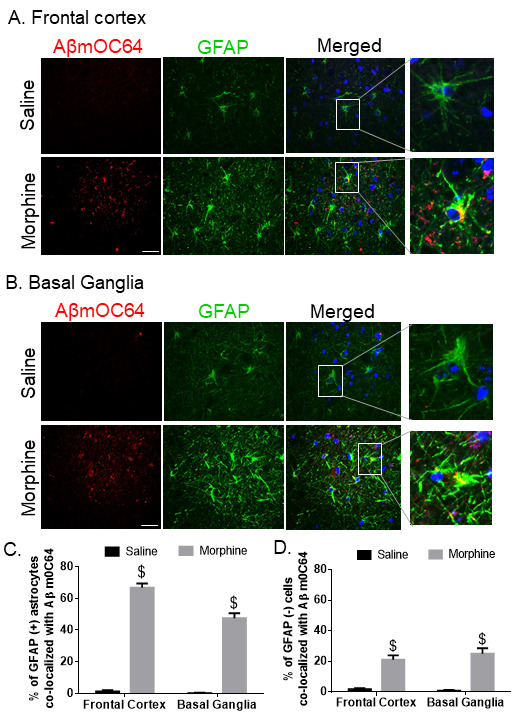
**Expression of astrocytic Aβ mOC64 in the FC and BG of morphine-dependent macaques**. Representative immuno-histochemistry photomicrographs showing differential expression of Aβ mOC64 protein in GFAP-positive astrocytes in the FC (A) and BG (B) of morphine-dependent macaques. Scale bar, 20 μm. Quantitative analysis of percent of GFAP positive (C) and negative astrocytes (D) colocalized with Aβ mOC64 protein in saline and morphine-dependent macaque. Ten fields from FC and BG/macaque were analyzed from *n*= 4 macaques per group. Data are presented as mean±SEM. Student *t* test was used to determine the statistical significance: $P< 0.001 versus saline. Abbreviations: Aβ, amyloid beta, GFAP, glial fibrillary acidic protein.

## RESULTS

### Morphine-dependent rhesus macaques exhibit brain region-specific amyloidosis

We first wanted to assess the expression of toxic amyloid proteins in brain homogenates of saline and morphine-dependent rhesus macaques by western blotting. As shown in Fig. 1A-B, there was significant up-regulation of toxic Aβ mOC64 ^(*P* < 0.01), APP ^(*P* < 0.01), and pro-inflammatory cytokine IL 1β protein *(*P* < 0.05) in the select brain regions- frontal cortex (FC) and basal ganglia (BG) of the morphine administered macaques versus the saline group. Further, qRT-PCR showed that in addition to protein levels, mRNA levels of the APP were also significantly upregulated in the frontal cortex $(*P* < 0.001) and basal ganglia *(*P* < 0.05) in the morphine group compared with the saline group (Fig. 1C).

### Immunohistochemistry of Aβ 1-42/ mOC64 expression in brain regions of morphine dependent macaques

**Figure 3.**
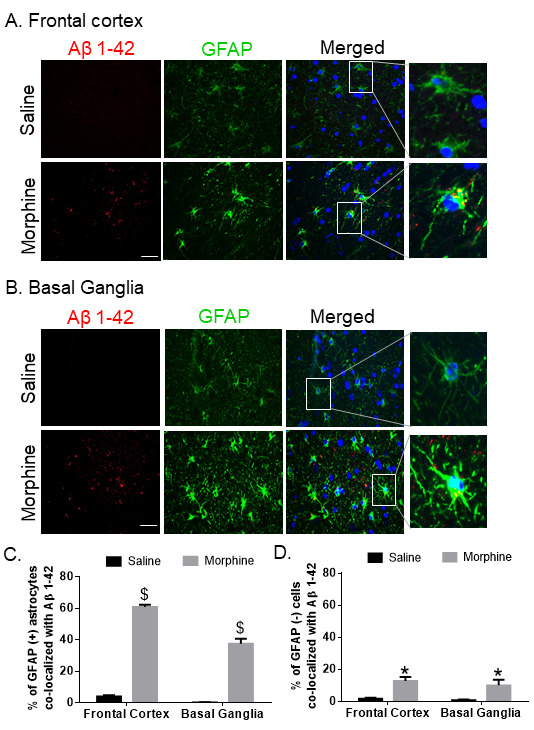
**Expression of astrocytic Aβ 1-42 in the FC and BG of morphine-dependent macaques**. Representative immunohistochemistry photomicrographs showing differential expression of Aβ 1-42 protein in GFAP-positive astrocytes in the FC (A) and BG (B) of morphine-dependent macaque. Scale bar, 20 μm. Quantitative analysis of percent GFAP positive (C) and negative (D) astrocytes colocalized with Aβ 1-42 protein in saline and morphine-dependent macaque FCs and BGs. Ten fields from FC and BG/macaque were analyzed from *n*= 4 macaques. Data are presented as mean±SEM. Student *t* test was used to determine the statistical significance: $P< 0.001, *P< 0.05 versus saline. Abbreviations: Aβ, amyloid beta, GFAP, glial fibrillary acidic protein.

Next, we sought to assess the presence of Aβ1-42 in glial fibrillary acidic protein (GFAP)-positive astrocytes in the brains of macaques. For this, brain sections from saline and morphine dependent macaques were co-immunostained for the presence of Aβ1-42/ Aβm0C64 and GFAP. As shown in Figs. 2-3, there was increased expression of both varieties of amyloids- Aβ1-42 and Aβm0C64 in the frontal cortex (FC) and basal ganglia (BG) of morphine dependent animals with a significant $(*P* < 0.001) colocalization with GFAP-positive astrocytes. Interestingly, we observed that GFAP-negative cells also showed increased expression of Aβ1-42 *(*P* < 0.05) as well as Aβm0C64 $(*P* < 0.001), indicating that cells other than astrocytes could also contribute to the process of amyloidosis. Quantitative analysis demonstrated that the percent of amyloids in GFAP negative cells was between 5% and 25% while that for GFAP positive cells was between 40% and 75%. We also found that several amyloids did not colocalize with the DAPI-nucleus, thereby indicating extracellular deposition of amyloids. Lower magnification images of Aβ1-42 and Aβm0C64 colocalizing with GFP positive astrocytes in the FC and BG of morphine dependent animals are shown in Supplementary Figs. 1-2.

**Figure 4.**
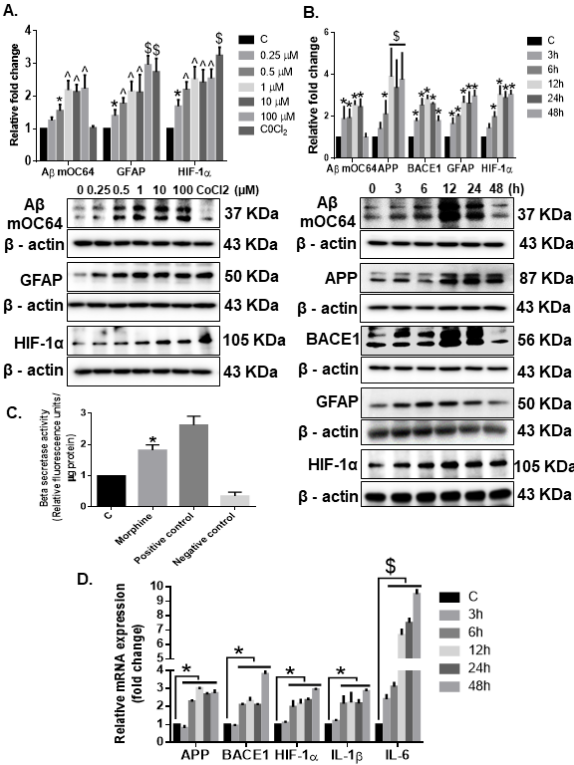
**Morphine-mediated amyloidosis and neuroinflammation in HPA**. Western blot analysis showing dose-dependent upregulation of Aβ mOC64, GFAP, and HIF-1α (A) and time-dependent upregulation of Aβ mOC64, BACE1, GFAP, and HIF-1α (B) in HPAs exposed to morphine at the indicated dose and time points. (**C**) Morphine (500 nM)-exposed HPAs showed increased β-secretase activity by spectrofluorometric analysis. (**D**) qPCR showing increased expression of APP, BACE1, HIF-1α, IL1-β, and IL1-6 mRNAs in HPAs exposed to morphine (500 nM) at the indicated time points. GAPDH was used as an internal control for mRNA expression. Data are presented as mean±SEM. One-way ANOVA followed by Bonferroni post hoc test was performed, $P< 0.001, ^P< 0.01, *P < 0.05 versus control. Abbreviations: APP, amyloid precursor protein, GFAP, glial fibrillary acidic protein, HIF-1α, hypoxia-inducible factor 1α, 1IL, interleukin, qPCR, quantitative polymerase chain reaction.

### Morphine exposure induces astrocytic amyloidosis

Based on our *ex vivo* findings, next we sought to explore the molecular mechanisms associated with morphine-induced astrocytic amyloidosis *in vitro*. HIF-1α [57] is a known inducer of BACE1 enzyme, which is crucial for sequential cleavage of Aβ from APP [20-22]. Herein human primary astrocytes (HPAs) were exposed to varying concentrations of morphine (0, 0.25, 0.5, 1, 10, 100 µM) for 24 hours followed by assessment of cell lysates for the expression of Aβ mOC64, GFAP, HIF-1α. As shown in Fig. 4A, morphine at 0.5 µM concentration significantly (*P < 0.05, ^*P* < 0.01, $*P* < 0.001) increased the expression of conformation-specific Aβ mOC64 and HIF-1α (CoCl_2_ was used as a positive control for HIF-1α expression) as well as GFAP in a dose-dependent manner-compared with control cells not exposed to morphine. It must be noted that this concentration (0.5 µM) of morphine is in keeping with the physiological levels of morphine found in the postmortem brain tissues of narcotic overdose individuals (200 ng/g of brain tissue) [58]. Since 0.5 µM morphine showed consistent and significant up-regulation of all the markers and closely matches with the concentration of morphine in the brains of opiate abusers, this concentration was chosen for future experimentation. Next, we wanted to determine the time-dependent (3, 6, 12, 24, 48 hours) expression of amyloid markers in astrocytes exposed to 0.5 µM of morphine. As shown in Fig. 4B, morphine (0.5 µM) significantly increased *(*P* < 0.05) the expression levels of Aβ mOC64, APP $(*P* < 0.001), HIF-1α, BACE1 and GFAP in a time-dependent manner in HPAs, with consistent up-regulation at 24 hours post-morphine exposure compared with control cells. Based on these findings, the optimal dose and time for morphine effect was 0.5 µM for 24 hours which was the chosen dose and time for all subsequent experiments. Morphine exposure was also found to significantly up-regulate *(*P* < 0.05) BACE1 activity in HPAs (Fig. 4C). Similar to findings in Fig 3A-C, morphine exposure also mediated induction of astrocytic amyloidosis by significantly upregulating *(*P* < 0.05) the mRNA expression of APP, BACE1 and HIF-1α as well as neuroinflammatory cytokines IL 1β and IL 6 $(*P* < 0.05) in HPAs exposed to morphine (Fig. 4D).

**Figure 5.**
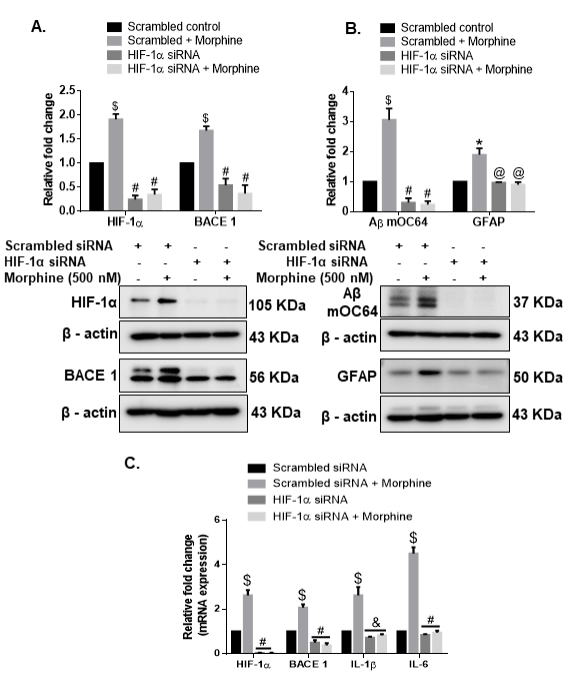
**Role of HIF-1α in morphine-mediated astrocytic amyloidosis**. Representative western blots showing the expressions of (A) HIF-1α and BACE1 and (B) Aβ mOC64 and GFAP in HPAs transfected with either HIF-1α siRNA or scrambled siRNA in the presence of morphine (500 nM). (**C**) qPCR showing expression of HIF-1α, BACE1, IL1-β, and IL1-6 RNA in HPAs transfected with either HIF-1α or scrambled siRNA. Data are presented as mean±SEM. One-way ANOVA followed by Bonferroni post hoc test was performed, $P< 0.001, *P < 0.05 versus control, #P< 0.001, &P< 0.01 versus scrambled siRNA+Morphine. Abbreviations: Aβ, amyloid beta, HIF-1α, hypoxia-inducible factor 1α, HIF-1α, hypoxia-inducible factor 1α, HPA, human primary astrocyte, IL, interleukin, qPCR, quantitative polymerase chain reaction, siRNA, small interfering RNA.

### HIF-1α regulated expression of toxic amyloid forms & neuroinflammation involves BACE1 in HPAs

**Figure 6.**
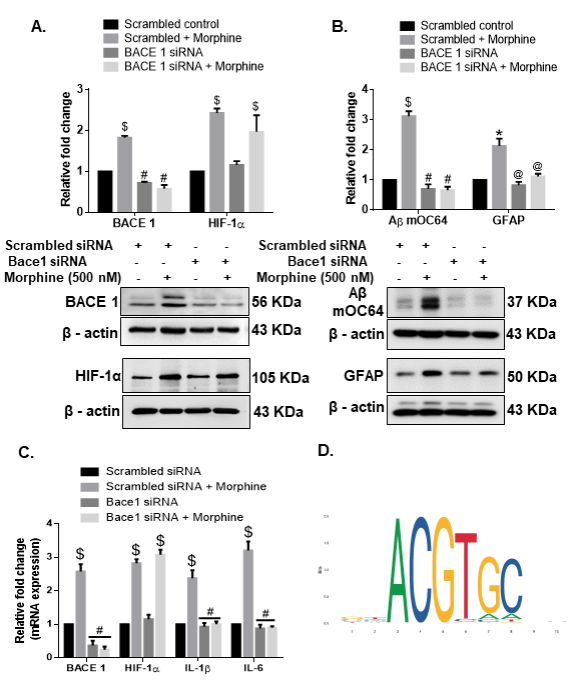
**Role of BACE1 in morphine-mediated astrocytic amyloidosis**. Representative western blots showing the expressions of (A) BACE1 and HIF-1α (B) Aβ mOC64 and GFAP in HPA transfected with either BACE1 siRNA or scrambled siRNA in the presence or absence of morphine (500 nM). (**C**) qPCR showing expression of BACE1, HIF-1α, IL1-β, and IL1-6 mRNA in HPAs transfected with either BACE1 or scrambled siRNA. (**D**) HIF-1α binding motif on the BACE1 promoter. Data are presented as mean±SEM. One-way ANOVA followed by Bonferroni post hoc test was performed, $P< 0.001, *P < 0.05 versus control, #P< 0.001, @P< 0.05 versus scrambled siRNA+ Morphine. Abbreviations: Aβ, amyloid beta, HIF-1α, hypoxia-inducible factor 1α, HPA, human primary astrocyte, IL, interleukin, siRNA, small interfering RNA.

Having determined that morphine can induce the expression of HIF-1α in HPAs, we next wanted to determine the role of HIF-1α in inducing the expression of BACE1, using the gene silencing approach. For this HPAs were transfected with either *HIF-1α* small interfering RNA (siRNA) or scrambled siRNA followed by exposure of cells to morphine (0.5 µM) and assessed for expression of HIF-1α, BACE1, IL 1β, IL 6 mRNA as well as intracellular expression of Aβ mOC64, BACE1, HIF-1α and GFAP. Scrambled siRNA-transfected HPAs followed by morphine exposure resulted in significant increases $(*P* < 0.001) in the mRNA expression of HIF-1α, BACE1, IL 1β, IL 6, as well in the protein levels of HIF-1α, BACE1, AβmOC64 and GFAP *(*P* < 0.05), compared with cells not exposed to morphine. HPAs transfected with HIF-1α siRNA in the presence of morphine exposure demonstrated significant decreases #(*P* < 0.001) in the expression of HIF-1α, BACE1, IL 1β &(*P* < 0.01), IL 6 RNA as well as decreased expression of HIF-1α, BACE1, Aβ mOC64, and GFAP proteins (Fig. 5A-C) compared with HPAs transfected with scrambled siRNA and exposed to morphine. To further validate these findings, we next silenced the BACE1 gene by transfecting the HPAs with BACE1 siRNA, followed by exposure of cells to morphine. As shown in Fig. 6A-C, in BACE1 siRNA-transfected HPAs in the presence or absence of morphine, demonstrated significantly decreased #(*P* < 0.001) expression of BACE1, IL 1β, IL 6 RNA and BACE1, Aβ mOC64 and GFAP @(*P* < 0.05) protein levels compared with morphine-exposed HPAs transfected with scrambled siRNA. Expression of HIF1-α in siRNA-transfected HPAs, however, remained unchanged in the presence of morphine. Gene silencing approach underpinned the role of HIF-1α as a regulator of BACE1 expression in presence of morphine. This was further confirmed by bioinformatic analysis which demonstrated presence of HIF-1α binding motif on the promoter of BACE1 (Fig. 6D).

### Increased expression of HIF-1α and BACE1 in the FC and BG of morphine-dependent rhesus macaques

**Figure 7.**
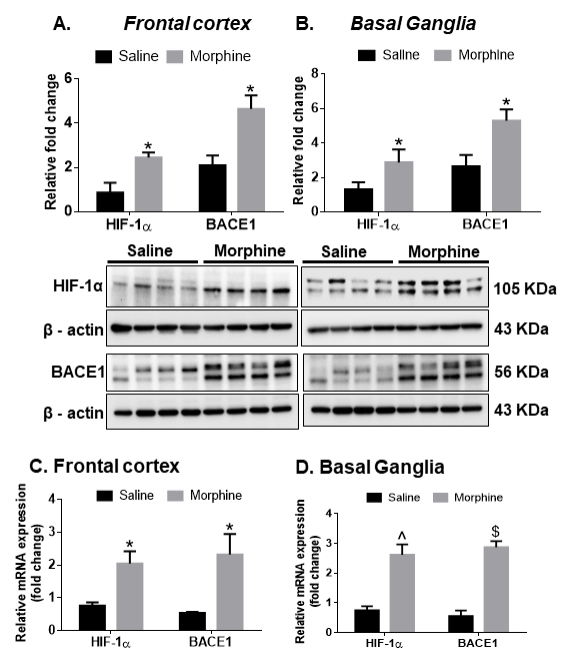
**Expression of HIF-1α and BACE1 in the brains of morphine-dependent macaques**. Representative western blots showing the expression of HIF-1α and BACE1 in the FC (A) and BG (B) of saline or morphine-dependent macaques. (**C**) qPCR demonstrating the expression of HIF-1α and BACE1 in the FC (C) and BG (D) of saline or morphine-dependent macaques. *n*= 4 macaques per group. Data are presented as mean±SEM. Student *t* test was used to determine the statistical significance between multiple groups: $P< 0.001, ^P< 0.01, *P< 0.05 versus saline. Abbreviations: HIF-1α, hypoxia-inducible factor 1α, qPCR, quantitative polymerase chain reaction.

Having determined the role of HIF-1α in mediating the expression of BACE1 in the presence of morphine, we next sought to assess the expression of both HIF-1α and BACE1 by western blotting in select brain regions (FC and BG) of morphine-dependent and saline control macaques. As shown in Fig. 7A-B, there was significant up-regulation *(*P* < 0.05) of HIF-1α and BACE1 proteins in both the FC and BG of morphine administered macaques compared with the saline controls. Furthermore, there was a significant upregulation in both HIF-1α *(*P* < 0.05) and BACE1 *(*P* < 0.05) mRNAs in the frontal cortex, HIF-1α ^(*P* < 0.01) and BACE1 $(*P* < 0.001) mRNAs in the basal ganglia of the morphine-dependent macaques compared with saline (Fig. 7C-D).

### Morphine administration induced the release of ADEVs carrying the amyloid cargoes in HPAs

**Figure 8.**
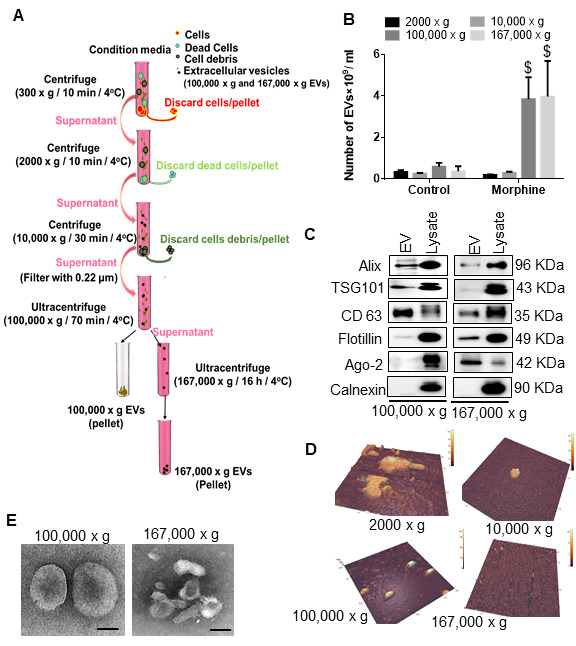
Characterization of astrocyte-derived EVs from human primary astrocytes. (**A**) Schematic representation of isolation protocol for extracellular vesicles (EVs) from HPAs. (**B**) Quantification of brain-derived EVs by ZetaView. (**C**) Representative western blot images showing expression of EV markers (Alix, TSG 101, CD 63, Flotiliin and Ago2). (**D**) Representative Atomic force microscopy (AFM) images of different fractions of EVs. (**E**) Representative Transmission Electron Microscopy (TEM) Images of different fractions of EVs. Data are presented as mean ± SEM. One-way ANOVA followed by Bonferroni post hoc test was performed, $P<0.001 versus control. Abbreviations: EVs, extracellular vesicles.

Herein we sought to determine whether ADEVs released from morphine-stimulated HPAs could also contain the amyloid cargoes. Astrocytes have been shown to produce considerable amounts of EVs [59] and were thus chosen for this study. For this we first isolated and characterized ADEVs from conditioned media of HPAs, using a differential ultracentrifugation procedure (Fig. 8A) [60, 61]. Purified EVs were quantified in Nanoparticle Tracking Analysis (NTA). As shown, the numbers of both 100,000 × g-exosomes (Control-ADEV 0.5975×10^9^ ± 0.1855×10^9^ and morphine exposed ADEV 3.850×10^9^ ± 1.060×10^9^) and 167,000 × g-exomeres (Control-ADEV 0.3725*10^9^ ± 0.2456*10^9^ and morphine exposed ADEV 4.475×10^9^ ± 1.580×10^9^) were significantly $(p< 0.001) increased in supernatants obtained from cells exposed to morphine (0.5 µM, 24h) compared with the controls (Fig. 8B). However, as expected, morphine treatment did not affect the release of 2000 x g and 10,000 x g non-EV particles compared to control group not treated with morphine. Immunoblotting of the EV lysates revealed the presence of exosomal markers - TSG101, CD63 and Alix for the 100,000 x g vesicles, and exomere markers - CD63, Flotillin and Ago-2 for the 167,000 xg exomeres (Fig. 8C). Additionally, immunoblotting using calnexin was also assessed to rule out contamination of ADEVs with the cell debris. Next, the EVs were further characterized by transmission electron microscopy (TEM) and atomic force microscopy (AFM) Fig. 8D-F and as presented in Table 1 demonstrated that EVs had an average diameter of 103 nm for exosomes, and 48 nm for exomeres (Table 1).

**Figure 9.**
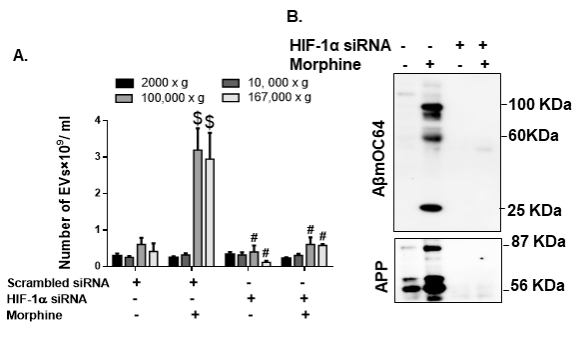
**Role of HIF-1α in morphine mediated EV release and cargoes**. (**A**) Quantitative analysis of EV numbers isolated from condition media of HPAs transfected with either scrambled siRNA or HIF-1α siRNA followed by exposure to morphine. (**B**) Representative western blot images showing protein levels of Aβ mOC64 and APP in HPAs transfected with either scrambled or HIF-1α siRNA followed by exposure to morphine. Data are presented as mean±SEM. One-way ANOVA followed by Bonferroni post hoc test was performed, $P< 0.001 versus control, #P< 0.001 versus scrambled siRNA+Morphine. *P < 0.05 versus control. Abbreviations: APP- amyloid precursor protein, Aβ- amyloid beta, HIF-1α- hypoxia-inducible factor, siRNA- small interfering RNA.

Based on our findings that morphine-mediated induction of HIF-1α regulated downstream amyloid production in HPAs, we next sought to assess the role of HIF-1α in morphine-mediated release of ADEVs. For this we used genetic silencing approach to abrogate the expression of HIF-1α in HPAs followed by assessment of ADEV numbers released by the HPAs. As shown in Fig. 9A, there was a significant decrease #(p< 0.001) in the numbers of ADEVs isolated from HIF-1α silenced HPAs compared with HPAs transfected with scrambled siRNA, in the presence of morphine. Additionally, morphine (0.5 µM, 24h) exposure also increased the packaging of various toxic forms of amyloid cargoes in the ADEVs (167,000 xg) (Fig. 9B). There were no traces of amyloids in ADEVs isolated from HIF-1α silenced cells.

**Table 1:** Quantitative analysis (width, height, volume) of different fractions of EVs by AFM.

	2,000 x g	10,000 x g	100,000 x g	167,000 x g
Width (nm)	148.1 ± 85	125.1 ± 47	103 ± 53	48 ± 6
Height (nm)	25.8 ± 12.3	18.9 ± 15	11.9 ± 10	10 ± 3.7
Volume (nm³)	149731 ± 116000	75726 ± 83213	45418+460000	9324 ± 3790

### Characterization of brain-derived EVs from saline- and morphine-dependent macaques

**Figure 10.**
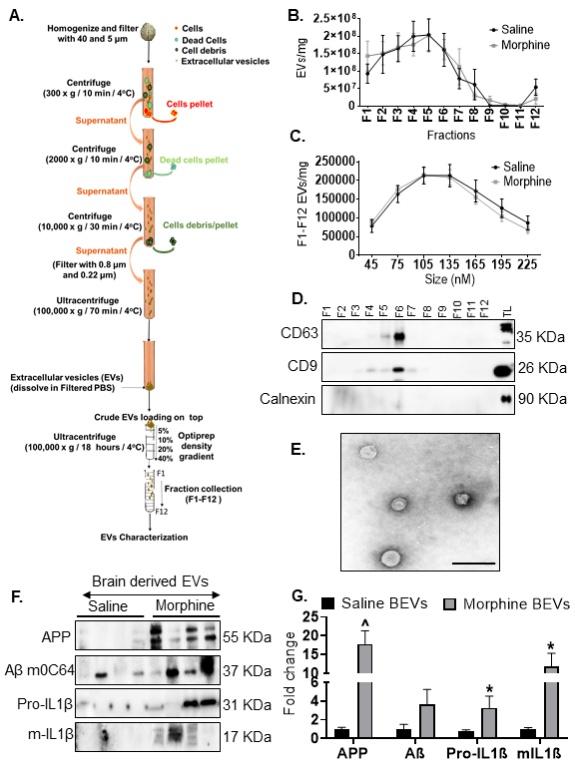
**Characterization of EVs from macaque brain**. (**A**) Schematic representation of isolation protocol for EVs from brain of macaques. (**B**) Quantification of EVs per mg brain tissue from different fractions (F1-F12) assessed by ZetaView. (**C**) Size distribution of EVs for the size range 45-225 nm (F1-F12) assessed by ZetaView. (**D**) Representative western blot images showing EV specific markers (CD63, CD9) in different fractions (F1-F12). (**E**) Topographic profiling of F4-F7 EVs using transmission electron microscopy (TM) under tapping mode revealed a heterogeneous population of spherical particles. (F, G) Representative western blot images and quantification (normalization with proteins in the EVs) for APP, AβmoC64, Pro-IL1β, m-IL-1β in brain derived EVs (F4-7) from saline or morphine-dependent macaques. *P< 0.05, ^P< 0.01 versus saline. Abbreviations: Aβ- amyloid beta, EVs- extracellular vesicles, IL- interleukin.

Herein we sought to assess whether brain derived EVs (BEVs) from morphine-dependent macaques also contained amyloid and neuroinflammatory cargoes. BEVs were isolated from the brains of macaques by enzymatic digestion, differential centrifugation, and purification using Optiprep density gradient centrifugation (schematic shown in Fig. 10A). We employed student *t*- test to determine the statistical significance between saline- and morphine- dependent macaques. Fig. 10B demonstrates the total number of EVs particles present in mg of the brain tissue in different EV fractions by NTA. Additionally, Fig. 10C demonstrates the size distribution of the EVs in different fractions from per mg of the brain tissue by NTA. Originally, the count of EVs were determined as present in per ml, and then converted to per mg of tissue. Size distribution assessed by NTA revealed EVs distribution in the 50-200 nm range, with maximum number of EVs between 100-135 nm size range. Interestingly, there was no change in numbers or size distribution of BEVs between the saline or morphine groups (Fig. 10B-C). More than 75% of BEVs ranging in size from 50-200 nm was present in fractions F3-6 (Fig. 10C-D). Western blotting of the BEVs demonstrated the presence of exosome-enriched proteins CD63 and CD9 specifically in F4-6 fractions and were negative for endoplasmic reticulum protein calnexin. Brain-lysate was used as a positive control for calnexin (Fig. 10D). The structure and diameter of the EVs was further confirmed by TEM analysis (Fig. 10E) and the same EV particles with TEM analysis quantification has been represented in Supplementary Fig. 3A. Loading equal numbers of BEVs/protein concentration for western blotting from both saline and morphine-dependent animals, demonstrated a significant increase in N-terminally cleaved APP ^(p< 0.01), AβmOC64 (trend of increase) as well as pro- and mature- IL-1β *(p< 0.05) in the BEVs from morphine-dependent macaques compared with saline group of macaques (Fig. 10F-G). Non-EV fractions from either of the macaque groups failed to exhibit the presence of the amyloid cargoes (Supplementary Fig. 3B). No significant change in protein concentration was observed between saline- and morphine- BEVs (Supplementary Fig. 3C).

## DISCUSSION

Chronic opiate use has been reported to cause multitude of cognitive deficits including impairment of attention, working memory, episodic memory [15, 16] as well as prospective memory [12]. On an average ~20-44% of the cancer patients taking morphine for pain control go on to develop cognitive decline [14]. Recent reports have demonstrated the presence of hyperphosphorylated Tau in the brains of chronic opiate users [24] and amyloid deposition was also observed in morphine-dependent rats [62]. Toxic amyloids have been reported to contribute to neurodegeneration and cognitive deficits observed in AD [63]. The present study for the first time demonstrates the role of astrocytes in regulating morphine-mediated amyloidosis with the involvement of HIF-1α-BACE1 axis, ultimately resulting in neuroinflammation. These findings were also validated in morphine-dependent macaques. Furthermore, we also report dissemination of morphine induced neuropathogenesis by ADEVs carrying the amyloid cargoes.

Astrocytes are an abundant cell type in the CNS, which are involved in several critical physiological functions in the brain such as neurogenesis, synaptogenesis, controlling the blood-brain barrier permeability and maintaining extracellular homeostasis [64-67]. Recent findings have demonstrated the role of astrocytes in morphine-mediated neuroinflammation via dysregulated autophagy pathway [50]. The present study demonstrates a unique role of astrocytes in morphine-mediated amyloid pathology which leads to neuroinflammation. We first examined astrocytic amyloidosis in the archival tissue sections of morphine dependent rhesus macaques *ex vivo*. In this study we have shown amyloid pathogenesis in both the frontal cortex (FC) and basal ganglia (BG), since these regions are involved in regulation of learning and memory [68, 69] as well as opiate addiction [70-72]. We observed increased amyloidosis as evidenced by immunostaining and western blotting for amyloids, specifically in the FC and BG of morphine-dependent compared with the saline administered macaques. These findings were also validated by increased mRNA expression of APP, BACE1 and HIF-1α RNA in the brains of morphine dependent macaques. Furthermore, we also validated our findings by immunostaining, wherein we demonstrated increased co-localization of GFAP positive astrocytes with the two varieties of neurotoxic amyloids- Aβm0C64 & Aβ 1-42, thus underscoring the role of astrocytes in morphine-induced amyloidosis. Quantitative analysis further confirmed that the percentage of GFAP+ astrocytes co-localizing with amyloids in these brain regions was significantly higher than the non-astrocytic cells, again validating the vital role of astrocytes in morphine-induced amyloid pathology, which, in turn, could be a key factor underlying cognitive deficits observed in opiate users. Interestingly, we also found higher expression of neuroinflammatory cytokines in the FC and BG of morphine dependent macaques [50]. Upregulation in the protein levels of IL-1β (in the FC and BG of morphine-dependent macaques) was also observed in the present study, thus indicating a positive correlation between morphine-induced amyloidosis and neuroinflammation.

**Figure 11.**
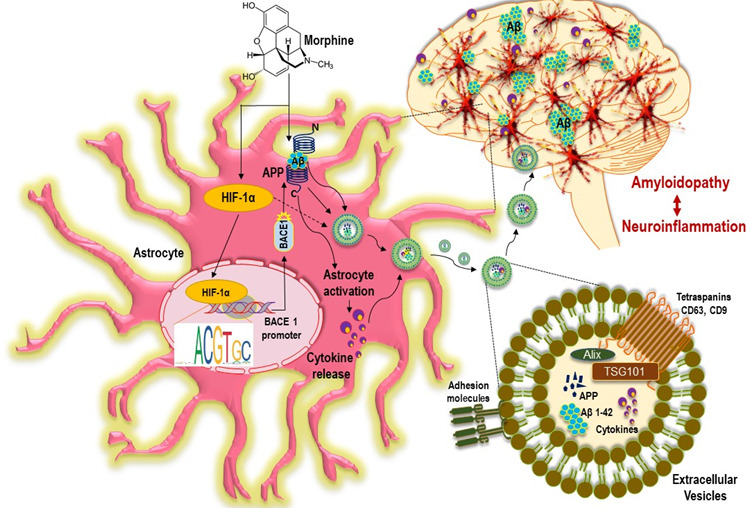
**Schematic representation on the role of morphine in extracellular vesicles mediated amyloidopathy and neuroinflammation: Exposure of HPAs to morphine increased the expression and accumulation of HIF-1α in the cytoplasm**. Nuclear translocation of HIF-1α, resulted in the binding of the former on the promoter of BACE1 gene, thus regulating the transcription of the BACE1 gene, which further cleaved the APP to release the Aβ. These neurotoxic Aβ, cause astrocyte activation and pro inflammatory milieu in the HPA. Additionally, morphine could induce the release of EVs via HIF-1α mediated pathway in HPAs. The various forms of amyloids were sorted into the EVs and are transported to different regions of brain resulting in widespread seeding of this amyloids. Additionally, amyloid induced cytokines were also released in the EVs leading to neuroinflammation. Accumulation of toxic Aβ in the brain regions further facilitate neuroinflammation and neurodegeneration, thus contributing to cognitive impairments in opiate users. APP: amyloid precursor protein; Aβ: amyloid beta; BACE1, β-site cleaving enzyme; HPA, human primary astrocyte, ADEV: astrocyte derived extracellular vesicles.

Our ex vivo findings were further validated *in vitro* using HPAs. Using these cells, we unraveled the molecular mechanism(s) underlying morphine-mediated astrocytic amyloidosis. In line with our *in vivo* findings, we found that exposure of HPAs to morphine, resulted in a dose- and time- dependent upregulation of the toxic amyloid forms, thereby underscoring the role of astrocytes in the process of amyloidosis. Additionally, we also observed an increase in BACE 1 activity in morphine exposed HPAs. Our results also showed that morphine increased the expression of APP, BACE1 translationally, while also inducing transcriptional activation of these genes.

Several studies have shown the involvement of amyloidopathy in ischemic brain damage [73, 74]. Furthermore, the involvement of HIF-1α has also been well-documented in amyloidosis associated with AD [57, 75]. Additionally, in our recent report we have demonstrated the role of HIF-1α, as a master regulator in the process of astrocytic amyloidosis following HIV-Tat exposure [26]. In keeping with our published findings with HIV Tat [26], morphine also induced amyloidosis and upregulated the expression of HIF-1α in HPAs both transcriptionally as well as translationally. HIF-1α has been reported to regulate BACE1 expression [26]. It must be noted that while in the non-CNS cells such as Lewis lung carcinoma cells [76] and cardiac myocytes [77] morphine reduces the expression of HIF-1α, however, in both morphine exposed astrocytes as well as in the brains of morphine dependent macaques there was a significant increase in the expression of HIF-1α. Our findings were also validated by bioinformatic analysis which showed HIF-1α binding motif on the promoter of BACE1, thus adding validation that HIF-1α can regulate the expression of BACE 1. Using gene silencing approaches we further confirmed HIF-1α as a master regulator and an upstream mediator that regulated the expression of BACE1 and generation of Aβ. It was also found that in addition to regulating amyloid production, the master regulator HIF-1α, also regulated astrocyte activation and ensuing neuroinflammation. One probable explanation for this could be the intracellular levels of amyloid that could upregulate neuroinflammation [78] or that proinflammatory cytokines could regulate the accumulation of the toxic amyloid forms [78-80].

Another finding of our study was the presence of amyloids in the morphine stimulated ADEVs. EVs are nanoscale double membrane bound vesicles that are released by diverse cell types in the microenvironment [81]. It has been well recognized that astrocytes can communicate via direct cell-cell interactions and gap junctions; however, in recent times, it is being increasingly appreciated that astrocytes can also communicate with other cells within the CNS, through the release as well as uptake of EVs by the recipient cells [82]. These EVs contain surface markers, as well as a biologically active cargo of metabolites, proteins, nucleic acids, and other molecules specific to their tissue (and cell) of origin, reflecting the physiological state of the tissue or the cells [83]. Several studies have shown that these EVs, particularly brain-secreted EVs (BEVs) can carry amyloids, specially in AD [84, 85] and can serve as biomarkers of disease progression. In the present study, for the first time we demonstrate that BEVs from morphine dependent macaques contain varieties of post-translationally modified Aβm0C64 (of different molecular weight) as well as APP, full length and the N-terminally cleaved APP (55 KDa), both of which are considered to be highly neurotoxic [86]. Additionally, we have found that the cytokines (IL-1β) which were upregulated in the brains of morphine dependent macaques were also released in the BEVs. Increased numbers of EVs carrying toxic amyloids could likely play a key role in seeding in the different brain regions, thereby leading to progression of disease pathogenesis as well as neurodegeneration and neuroinflammation. Additionally, the cytokines released via the BEVs could also add to the neuroinflammatory burden in the brain. As discussed earlier, several studies have reported a close link between amyloids and neuroinflammation [78-80], such that both the released amyloids and the neuroinflammatory factors can lead to progressive neurodegeneration and neuroinflammation via an inter-related cascade. It was intriguing to note that although the BEVs from saline and morphine dependent macaques did not alter in numbers, size distribution and protein content, there was increased amyloid and neuroinflammatory cargo in morphine BEVs compared to the saline group BEVs, thus leading to the speculation that there was increased neurotoxic protein cargo burden in the morphine dependent macaques. Our *in vitro* mechanistic findings also showed that the numbers of ADEVs (both 100,000 × g and 167,000 × g vesicles) from morphine exposed astrocytes were significantly higher than the control group. Previous studies by our group as well as others have shown that morphine can increase the release of 100,000×g ADEVs [43], however, this is the first study demonstrating the presence of 167,000 × g ADEVs also in morphine-induced neuropathogenesis. This 167,000 × g particles differed in height, diameter and volume as well as protein markers (Argonuate 2 rich) compared with the 100,000 × g ADEVs (Alix, TSG101, CD63 rich). Our findings showed that the 167,000 × g morphine-ADEVs, but not the 100,000 × g morphine-ADEVs, had high levels of amyloid and IL-1β cargoes. Interestingly, the release of ADEVs as well as the cargoes, were regulated by HIF-1α as demonstrated by gene silencing studies. HIF-1α has been also shown earlier to regulate EV release [87], however, this is the first report demonstrating the regulation of amyloid and neuroinflammatory cargoes in the ADEVs by HIF-1α. ADEVs have been used in the *in vitro* study, since our study was primarily focused on assessing the role of astrocytes in morphine-induced amyloidosis. For the *in vivo* study we focused on total brain EVs to validate our hypothesis. We do acknowledge that this does not allow us to dissect the role of EVs from a particular cell type in morphine-induced amyloidosis. Considering the novel role of morphine in amyloidosis, it is important to show the overall effects of morphine on total brain cells and brain cell derived EVs.

In summary, our studies implicate that morphine can induce the expression of transcription factor- HIF-1α, which upon being translocated into the nucleus binds to the BACE 1 promoter to induce its synthesis. Increased level of BACE1 protein and its activity leads to cleavage of the transmembrane protein APP (upregulated independently by morphine) to its toxic Aβ forms, which can cause activation of astrocytes leading to generation of pro-inflammatory cytokines (Fig. 11). Findings by Kalman et. al., showed that morphine administration exhibited a trend of increased membrane bound APP compared with the control rats in the cortex [62]. While the focus of the current study is on astrocytic amyloidosis, the contribution of neuronal amyloidosis cannot be overlooked, however, based on our *in vivo* data, it appears that the contribution of astrocytes to the process of amyloidosis is higher than that of the non-astrocytic CNS cells. Our findings also demonstrate that the toxic isoforms of amyloids are released by EVs, which, in turn, could be re-distributed and seeded to different sites and cell types within the brain, leading to widespread neurodegeneration and neuroinflammation. Such an amyloid-mediated neurodegeneration as well as neuroinflammation, could contribute to the cognitive deficit observed in long-term opiate users, and could be targeted for future development of adjunctive therapies for chronic opiate users.

## Supplementary Materials

The Supplemenantry data can be found online at: www.aginganddisease.org/EN/10.14336/AD.2021.0406.


