# Supplementary material for: Astrocytes & Astrocyte derived Extracellular Vesicles in Morphine Induced Amyloidopathy: Implications for Cognitive Deficits in Opiate Abusers
Source: Aging Dis. 2021 Sep 1;12(6):1389–408. doi: 10.14336/AD.2021.0406 (PMC8407877; doi:10.14336/AD.2021.0406)
Supplement: Supplementary file 1 [file AD-12-6-1389-s.pdf]

SUPPLEMENTARY DATA

Astrocytes & Astrocyte derived Extracellular Vesicles in Morphine Induced Amyloidopathy: Implications for Cognitive Deficits in Opiate Abusers

Susmita Sil*, Seema Singh, Divya T. Chemparathy, Ernest T. Chivero, Lila Gordon, Shilpa Buch*

© 2021. Sil S et al. Published online at http://www.aginganddisease.org/EN/10.14336/AD.2021.0406

SUPPLEMENTARY DATA

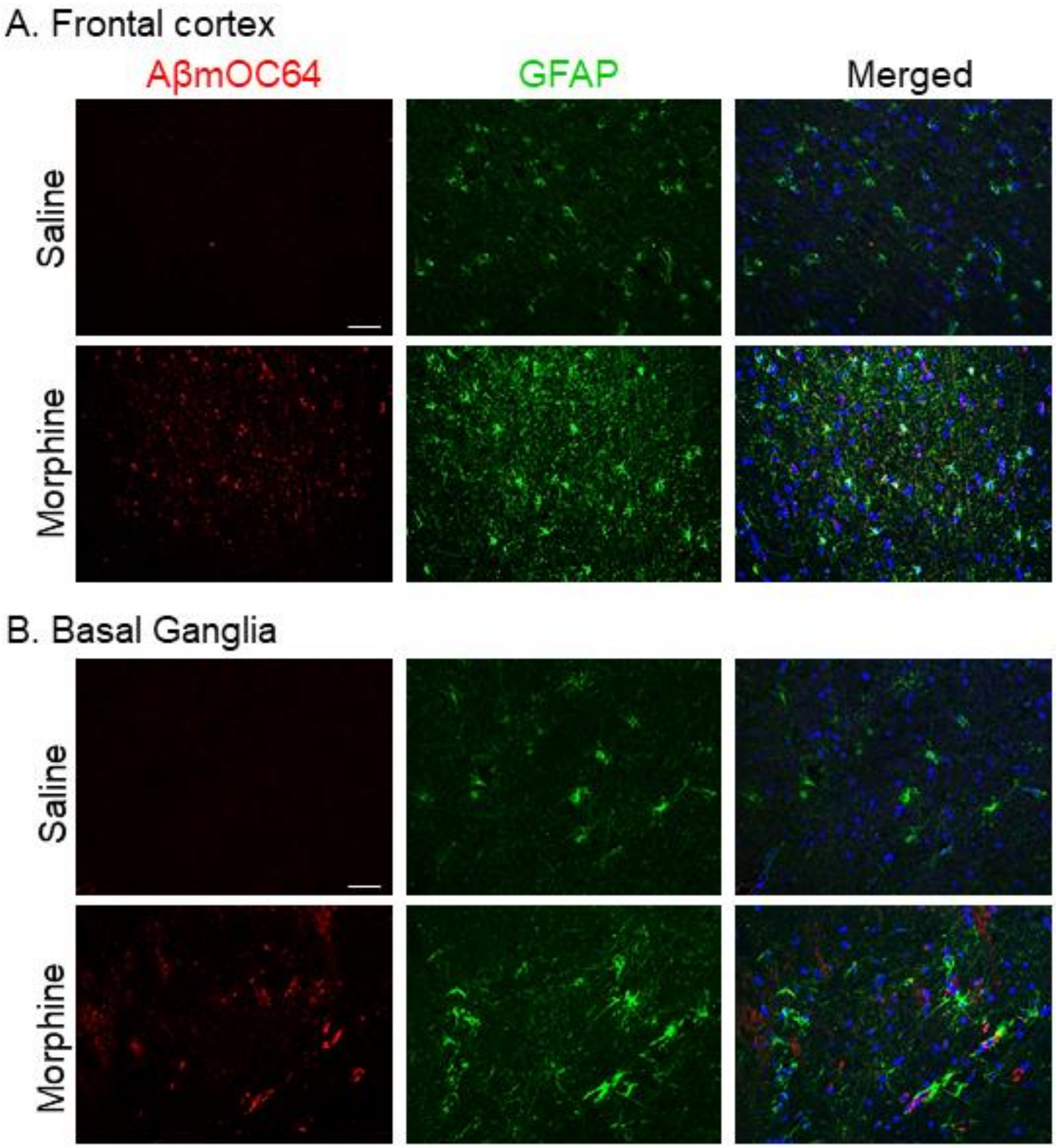

Supplementary Figure 1. Expression of Aβ mOC64 in the FC and BG of morphine-dependent macaques. Representative immunohistochemistry photomicrographs showing differential expression of Aβ mOC64 protein in GFAP+ astrocytes in morphine dependent macaque FCs (A) and BGs (B). Scale bar, 10 µm. *n*= 4 macaques/ group. Abbreviations: Aβ- amyloid beta, GFAP- glial fibrillary protein.

© 2021. Sil S et al. Published online at http://www.aginganddisease.org/EN/10.14336/AD.2021.0406

SUPPLEMENTARY DATA

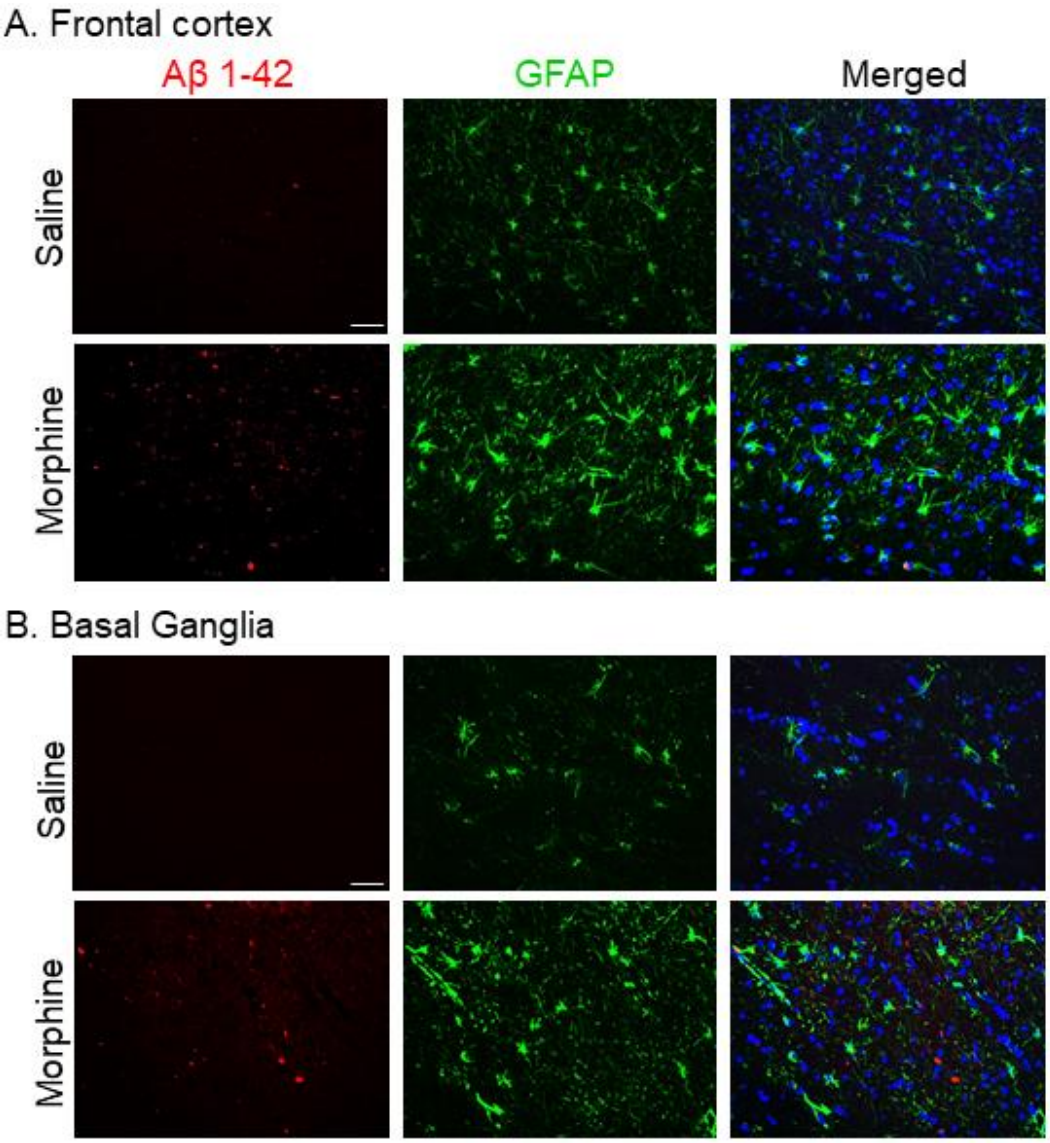

Supplementary Figure 2. Expression of Aβ 1-42 in the FC of morphine-dependent macaques. Representative immunohistochemistry photomicrographs showing differential expression of Aβ mOC64 protein in GFAP+ astrocytes in morphine dependent macaque FCs (A) and BGs (B). Scale bar, 10 μm. *n*= 4 macaques/ group. Abbreviations: Aβ- amyloid beta, GFAP- glial fibrillary acidic protein.

© 2021. Sil S et al. Published online at http://www.aginganddisease.org/EN/10.14336/AD.2021.0406

A.

B.

C.

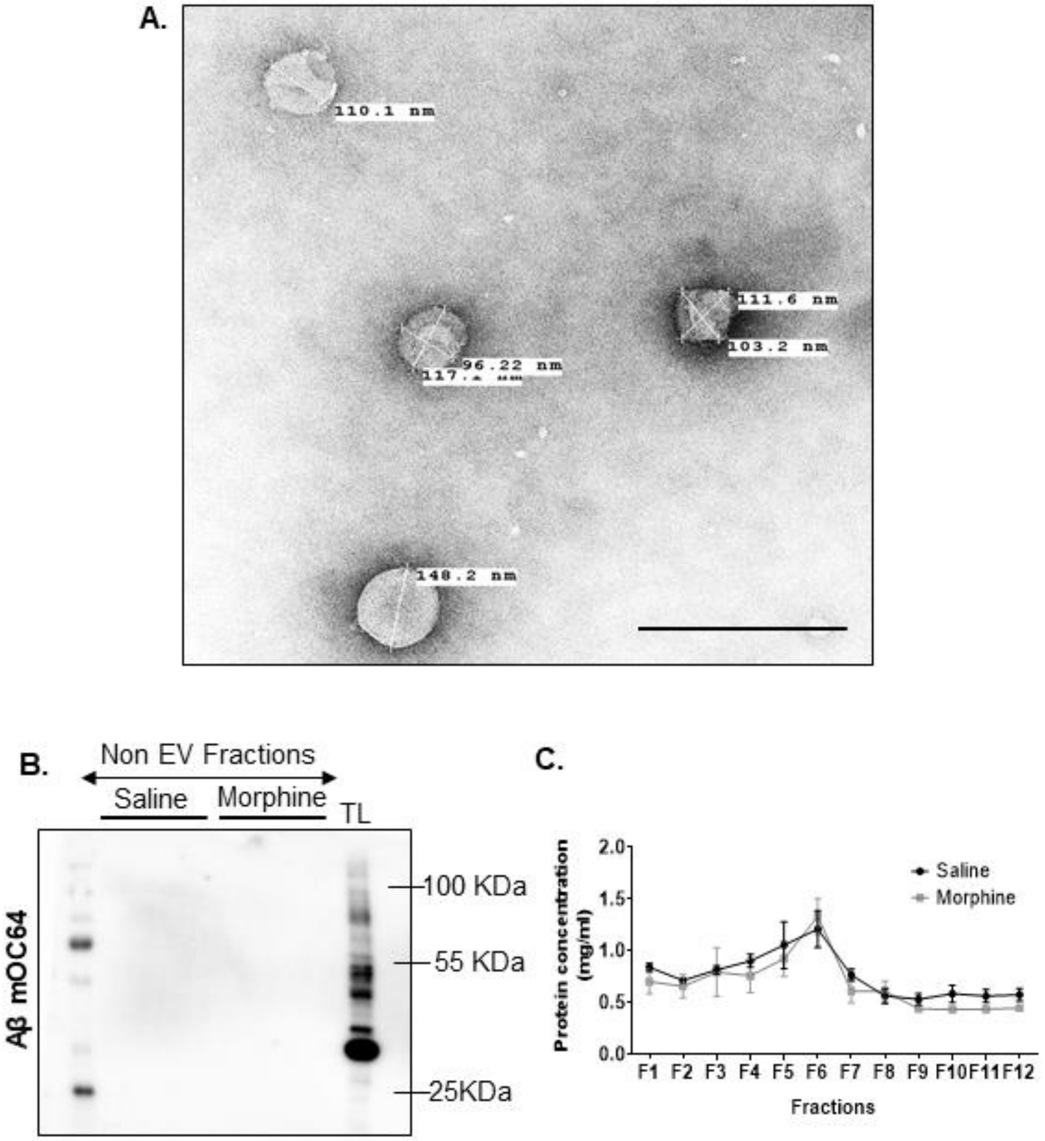

Supplementary Figure 3. Chracterization of brain-derived EVs from macaques. (A) Topographic profiling of F4-F7 EVs using transmission electron microscopy (TEM) under tapping mode revealed a heterogeneous population of spherical particles. (B) Representative western blot images for Aβm0C64 from non-EV fractions from saline and morphine dependent macaques. (C) Protein concentration in EVs from F1-F12 assessed by ZetaView. *n*= 4 macaques/ group.

© 2021. Sil S et al. Published online at http://www.aginganddisease.org/EN/10.14336/AD.2021.0406
